# Complex transmission epidemiology of neglected Australian arboviruses: diverse non-human vertebrate hosts and competent arthropod invertebrate vectors

**DOI:** 10.3389/fmicb.2024.1469710

**Published:** 2024-09-04

**Authors:** Andrew W. Taylor-Robinson

**Affiliations:** ^1^College of Health Sciences, VinUniversity, Hanoi, Vietnam; ^2^Center for Global Health, Perelman School of Medicine, University of Pennsylvania, Philadelphia, PA, United States; ^3^College of Health and Human Sciences, Charles Darwin University, Casuarina, NT, Australia

**Keywords:** arbovirus, neglected, transmission, arthropod, vector, reservoir host, enzootic, Australia

## Abstract

More than 75 arboviruses are indigenous to Australia, of which at least 13 are known to cause disease in humans. Alphaviruses are the most common arboviruses, notably including Ross River and Barmah Forest viruses, which contribute a significant public health and economic burden in Australia. Both can cause febrile illness with arthritic symptoms. Each circulates nationally across diverse climates and environments, and has multi-host, multi-vector dynamics. Several medically important flaviviruses also circulate in Australia. Infection with Murray Valley encephalitis or Kunjin viruses is less common but is associated with brain inflammation. Key research priorities for Australian arboviruses aim to understand clinical manifestations, develop timely diagnostics, and identify transmission cycles that permit the maintenance of arboviruses. While these can now be answered for a handful of notifiable alpha- and flaviviruses there are others for which non-human vertebrate hosts and competent arthropod invertebrate vectors are still to be identified and/or whose role in transmission is not well understood. One or more of these ‘neglected’ arboviruses may be the causative agent of a proportion of the many thousands of fever-related illnesses reported annually in Australia that at present remain undiagnosed. Here, what is known about enzootic cycling of viruses between arthropod vectors and mammalian and avian reservoir hosts is summarised. How and to what extent these interactions influence the epidemiology of arbovirus transmission and infection is discussed.

## 1 Introduction

Arthropod-borne viruses, commonly known as arboviruses, are a polyphyletic group of RNA viruses that circulate between different arthropod vectors (insects, usually mosquitoes, but also midges, sand flies and black flies; and arachnid ticks) and human and non-human vertebrate hosts (Franz et al., 2015). While these are predominantly mammals and birds the possible role of reptiles and amphibians in arbovirus transmission cycles has been discussed (Bosco-Lauth et al., 2018). These viruses are typically transmitted through the bite of an infectious arthropod vector, which acquires the virus by feeding on a viraemic vertebrate host (Lequime et al., 2016).

Arboviruses are significant health threats in tropical and sub-tropical regions with 3.9 billion people at risk, leading to an estimated disease burden of 300,000 to 5 million disability-adjusted life years lost annually (Labeaud et al., 2011). Arboviral infection can cause disease in vertebrates but does not trigger significant pathology in arthropods (Franz et al., 2015). While some human arboviral infections are asymptomatic or present with a mild influenza-like illness, arboviral pathogens causing serious illness ranging from rash and arthritis to encephalitis and haemorrhagic fever are an increasing threat to global health security (Wilder-Smith et al., 2017). The most striking illustration of this is the worldwide advance of dengue over the last 70 years (Gyawali et al., 2016a), while Japanese encephalitis virus (JEV) causes the majority of viral encephalitis cases in Asia (World Health Organization, 2019). Moreover, recent epidemics of chikungunya (CHIKV) and West Nile virus infection, plus the transcontinental spread of Zika virus (ZIKV), exemplify the growing risk posed by previously obscure pathogens and hence justify the concern over such emerging arboviruses (Gyawali et al., 2016b).

The interactions between invertebrate arthropod vectors and vertebrate hosts play a crucial role in the transmission and maintenance of arboviruses in nature (Kuno and Chang, 2005). Arthropod vectors serve as both the primary natural reservoirs and the means of transmission for arboviruses, while vertebrate hosts can act as amplifiers of viral replication (Kuno and Chang, 2005). The transmission cycle between these hosts allows for the persistence and spread of arboviruses in the environment. Arboviruses generally establish lifelong infection in vectors but exhibit transient infection of variable magnitude and duration in vertebrate hosts (Althouse and Hanley, 2015). Host factors such as tissue barriers, immune responses, genetic diversity, and replication dynamics all contribute to the complex dynamics of arbovirus transmission. Important ecological factors include population abundance, vector-host contact rate, and host migratory and other behaviours.

Only by fully understanding interactions between vectors and hosts can arbovirus transmission be controlled and/or prevented. Nowhere is this of more relevance than Australia, where more than 75 arboviruses that are indigenous to the country have been identified (Centers for Disease Control and Prevention, 2024). The alphaviruses Ross River (RRV) and Barmah Forest (BFV), and the flavivirus Murray Valley encephalitis (MVEV), are established as causative agents of debilitating diseases (e.g., Fraser, 1986), each of which may be detected by both antibody-based recognition and nucleic-acid amplification. However, for most of the remaining arboviruses (e.g., Alfuy [ALFV], Edge-Hill [EHV], Kokobera (KOKV], Sindbis [SINV], and Stratford [STRV]), that are or may be associated with pathology in humans, including some undifferentiated febrile illnesses routine tests are not available to diagnose infection (Gyawali et al., 2017a; Gyawali et al., 2019a). Prominent among public health challenges in parts of Australia north of the Tropic of Capricorn, as well as occasionally in more southerly latitudes, are so-called ‘neglected’ Australian arboviruses. Some of these viruses cause acute undifferentiated febrile illness, for which over half of all cases that occur annually in Australia are not diagnosed (Susilawati and McBride, 2014). Instigating a rigorous identification program would reduce the possibility of significant outbreaks of these indigenous arboviruses at a time when population growth accelerates across regional Australia, thereby bringing humans into closer proximity of native wildlife and vectors (Gyawali et al., 2017a).

Until very recently, JEV was limited to Far North Australia (Torres Strait Islands and Tiwi Island). During the hot, wet summers of 2021-22 and 2022-23, however, there was a dramatic geographical expansion of JEV across central and southern Australia (Pendrey and Martin, 2023). Of 45 clinical cases (35 laboratory-confirmed and 10 suggestive epidemiologically and/or symptomatically), there were seven deaths (Yakob et al., 2023). Given that local transmission occurred over two consecutive mosquito seasons, it is likely that JEV is now established endemically on the Australian mainland, placing as much as 750,000 people, 3% of the national population, at risk of JEV (Yakob et al., 2023). While sporadic cases of the closely related MVEV are largely confined to Northern Australia, major outbreaks were reported across southern and eastern regions of the country in 1951 (45 cases), 1974 (58 cases) and 2011 (17 cases) (Selvey et al., 2014a). In early 2023, six cases of MVEV infection were notified in the south-eastern state of Victoria, three of which were fatal (Braddick et al., 2023).

The concurrent (re-)emergence and co-circulation of JEV with MVEV (McGuinness et al., 2023) highlights key knowledge gaps in vector ecology, transmission dynamics and intervention efficacy. Integral to tailoring a control and prevention strategy to suit all Australian arboviruses is a better understanding of the interactions between their arthropod vectors and vertebrate hosts, which underpins their transmission epidemiology. While the number of arthropods from which these viruses have been recovered is considerable, less is known about the non-human vertebrate hosts that may be involved in their environment cycling or in the biting preferences of different mosquito species for these different reservoir hosts (Gyawali et al., 2019b; Gyawali et al., 2020). Information on the epidemiology and ecology of most of the neglected arboviruses is sketchy but they are known, or at least assumed, to be largely maintained in zoonotic cycles rather than exclusively by human-to-human transmission.

## 2 Transmission cycles of arboviruses

The transmission cycle of an arbovirus is determined by virus-vector host interactions. Arboviruses are transmitted between hosts by their arthropod vectors. The transmission cycle starts when an arthropod feeds on viraemic blood. Arboviruses can establish infection in the midgut epithelial cells of the arthropod vector and subsequently replicate in various tissues, including the salivary glands, enabling transmission to occur subsequently when the vector takes a blood meal (Lequime et al., 2016). Many arboviruses (e.g., MVEV, KUNV and JEV) have complex transmission cycles that include multiple host and vector species in maintenance and spillover (Kuno and Chang, 2005). Some hosts develop sufficiently high viraemias to infect susceptible vectors that feed on them while others do not. Failure to develop a viraemia sufficient to infect a vector does not mean that the host will not develop clinical symptoms. Cycles of transmission may involve only arthropods and humans (e.g., epidemic cycle of dengue virus, DENV, and RRV) or only non-human vertebrates and vectors (e.g., Akabane virus) or there may be transmission of viruses between human and non-human hosts (zoonoses, e.g., BFV, MVE, RRV). Most Australian arboviruses are zoonotic and maintain enzootic cycles involving birds and mammals as reservoir hosts (Go et al., 2014; Russell and Kay, 2004). In this cycle, the virus is continuously maintained in nature and may or may not cause disease in the enzootic host. Infections and epidemics in human populations can arise from direct spill-over of these enzootic and epizootic (exploiting domestic animals, e.g., JEV) cycles when amplification achieves a viraemia high enough for transmission (Weaver and Barrett, 2004).

## 3 Arboviruses and their vertebrate hosts

### 3.1 Mammals

Vertebrates can be considered as a possible reservoir for an arbovirus when a minimum of three commonly used criteria are met: viraemia; virus isolation; and relatively high antibody titres. While the presence of serum antibody against arboviruses in a host does not, *per se*, prove that an animal has been viraemic or involved in virus transmission as a reservoir, serological surveys do provide information about which animals might be involved in the transmission cycles of arboviruses (Table 1).

**TABLE 1:** Association of Australian arboviruses in domestic and native animals.

Vertebrate	Location	RRV	BFV	SINV	ALFV	EHV	KOKV	KUNV	MVEV	STRV	Reference
Cow	New South Wales	+	N/T	N/T	N/T	N/T	N/T	N/T	N/T	N/T	Cloonan et al., 1982
	Eastern Queensland	N/T	N/T	N/T	N/T	N/T	N/T	N/T	+	N/T	Doherty et al., 1964
	Brisbane	+	N/T	+	N/T	N/T	N/T	N/T	N/T	N/T	Doherty et al., 1966
	Mitchell River Mission	+	N/T	+	+	+	+	+	+	N/T	Doherty et al., 1971
	Northern Australia	+	N/T	N/T	N/T	N/T	N/T	N/T	+	N/T	Doherty et al., 1973
	Queensland	+	N/T	+	N/T	+	+	+	+	+	Sanderson, 1969
	New South Wales	+	+	N/T	N/T	N/T	N/T	N/T	N/T	N/T	Vale et al., 1991
Horse	Eastern Australia	N/T	N/T	N/T	N/T	N/T	N/T	N/T	+	N/T	Anderson et al., 1952
	Victoria	+	N/T	N/T	N/T	N/T	N/T	N/T	N/T	N/T	Azuolas, 1997
	South coast New South Wales	+	N/T	N/T	N/T	N/T	N/T	N/T	N/T	N/T	Cloonan et al., 1982
	Gympie, Brisbane outer suburb	+	N/T	+	N/T	N/T	N/T	N/T	+	N/T	Doherty et al., 1966
	Mitchell River Mission	+	N/T	N/T	N/T	N/T	N/T	N/T	N/T	N/T	Doherty et al., 1971
	Brisbane	+	+	N/T	N/T	N/T	N/T	N/T	N/T	N/T	Kay et al., 2007
	New South Wales	+	+	N/T	N/T	N/T	N/T	N/T	N/T	N/T	Vale et al., 1991
Dog	North and south Queensland	N/T	N/T	N/T	N/T	N/T	N/T	N/T	+	N/T	Doherty et al., 1964
	Redcliffe, Brisbane outer suburb	+	N/T	+	N/T	N/T	N/T	N/T	N/T	N/T	Doherty et al., 1966
	Mitchell River Mission	+	N/T	+	+	+	+	+	+	N/T	Doherty et al., 1971
	Brisbane	+	+	N/T	N/T	N/T	N/T	N/T	N/T	N/T	Kay et al., 2007
Cat	Brisbane	+	+	N/T	N/T	N/T	N/T	N/T	N/T	N/T	Kay et al., 2007
Wallaby	North and south Queensland	N/T	N/T	N/T	N/T	N/T	N/T	N/T	+	N/T	Doherty et al., 1964
	Gympie, Brisbane outer suburb	+	N/T	+	N/T	N/T	N/T	N/T	N/T	N/T	Doherty et al., 1966
	Mitchell River Mission	+	N/T	+	+	+	+	+	+	N/T	Doherty et al., 1971
	New South Wales	+	^_^	N/T	N/T	N/T	N/T	N/T	N/T	N/T	Vale et al., 1991
Kangaroo	North and south Queensland	N/T	N/T	N/T	N/T	N/T	N/T	N/T	+	N/T	Doherty et al., 1964
	Mitchell River Mission	+	N/T	+	+	+	+	+	+	N/T	Doherty et al., 1971
	Western Queensland	+	N/T	+	N/T	N/T	N/T	N/T	N/T	N/T	Doherty et al., 1966
	New South Wales	+	+	N/T	N/T	N/T	N/T	N/T	N/T	N/T	Vale et al., 1991
Fruit bat	Innisfail, north Queensland	^_^	N/T	^_^	N/T	N/T	N/T	N/T	N/T	N/T	Doherty et al., 1966
	Mitchell River Mission	+	N/T	^_^	+	^_^	+	+	+	N/T	Doherty et al., 1971
	Brisbane	+	^_^	N/T	N/T	N/T	N/T	N/T	N/T	N/T	Kay et al., 2007
Rat	North and south Queensland	N/T	N/T	N/T	N/T	N/T	N/T	N/T	^_^	N/T	Doherty et al., 1964
	Northeast Queensland	+	N/T	+	N/T	N/T	N/T	N/T	N/T	N/T	Doherty et al., 1966
Wild pig	Mitchell River Mission	+	N/T	^_^	^_^	^_^	+	+	+	N/T	Doherty et al., 1971
Common brushtail possum	Eastern Australia	N/T	N/T	N/T	N/T	N/T	N/T	N/T	+	N/T	Anderson et al., 1952
	All Australian states & territories	+	^_^	+	N/T	N/T	N/T	^_^	+	N/T	Azuolas, 1997
	Mitchell River Mission	+	N/T	^_^	N/T	N/T	N/T	N/T	+	N/T	Doherty et al., 1971
	Brisbane	+	+	N/T	N/T	N/T	N/T	N/T	N/T	N/T	Kay et al., 2007
Bandicoot	Northeast Queensland	+	N/T	+	N/T	N/T	N/T	N/T	+	N/T	Doherty et al., 1966

*Virus name abbreviations*: RRV, Ross River; BFV, Barmah Forest; SINV, Sindbis; ALFV, Alfuy; EHV, Edge Hill; KOKV, Kokobera; KUNV, West Nile virus Kunjin strain; MVEV, Murray Valley encephalitis; STRV, Stratford. N/T, not tested; +, antibodies detected; ^_^, antibodies not detected. Mitchel River Mission in Far North Queensland is now named Kowanyama.

#### 3.1.1 Marsupials

Macropods (notably, kangaroos and wallabies) are reservoirs and likely focal hosts of RRV. Laboratory infections and transmission of RRV from different kangaroos species to mosquitoes (Doherty et al., 1964; Doherty et al., 1966; Kay and Aaskov, 1989; Kay et al., 1986; Lindsay et al., 2005; Potter et al., 2014), isolation of RRV from agile wallabies (*Macropus agilis*) at Mitchell River Mission in Cape York Peninsula, Far North Queensland (Doherty et al., 1971), and viraemias of maximum titre of 4.6–5.6 suckling mouse intracerebral inoculation (SMIC) LD_50_/mL for between 4 and 6 days after introduction of RRV by the bite of infected mosquitoes (Kay et al., 1986) to Eastern grey kangaroos (*Macropus giganteus*) and agile wallabies have strongly suggested that marsupials are suitable hosts for RRV. While experimental studies of infection of macropods with all contemporary arboviruses have not been undertaken, one study reported titres of MVEV in eastern grey kangaroos as high as 10^3^ SMIC LD_50_/mL up to 6 days following the bite of an infected mosquito (Kay et al., 1986). Eastern grey kangaroos are terrestrial and widespread throughout eastern Australia, from Cape York to Victoria (Kay et al., 1985a). In Western Australia, the equivalent macropod is the closely related Western grey kangaroo (*Macropus fuliginosus*), which recent preliminary research (based on antibody detection) suggests is a common host for RRV and BFV (Gyawali et al., 2020). Common brushtail possums (*Trichosurus vulpecula*), a semi-arboreal marsupial, abound in the forest and urban areas throughout the eastern, northern, and south-western regions of Australia (How and Hillcox, 2000), and have been found to produce high titre viraemias following the bite of RRV-infected mosquitoes (Boyd et al., 2001), suggesting they may play a role in the urban transmission of RRV. Brushtail possums also generate mild viraemia for JEV (Daniels et al., 2000).

#### 3.1.2 Ungulates

The epidemic spread of RRV infection across the Western Pacific region in 1979–80 (Aaskov et al., 1981; Rosen et al., 1981; Tesh et al., 1981), and presence of historical antibody of RRV in animal sera collected from the south Pacific (Togami et al., 2020) have demonstrated endemic transmission of RRV in the absence of marsupial reservoirs. Serological studies have detected anti-RRV antibodies in domestic ungulates (large mammals with hooves), including cows and horses (Table 1). Comprehensive experimental studies of infection of cattle and horses with all arboviruses have not been undertaken. However, when cattle were infected with MVEV using orally infected *Culex annulirostris*, no viraemia was detected (Kay et al., 1985b). Similarly, when horses were infected with RRV by intravenous injection or the bite of infected *Cx. annulirostris*, virus could not be recovered in cell culture when sera from infected horses were cultured. Yet, the viraemia was sufficient to infect laboratory mosquitoes when *Ae. vigilax* and *Cx. annulirostris* were fed on the infected horses (Kay et al., 1987; Ryan et al., 1997). The presence of anti-RRV antibodies in horses has led some arbovirologists to propose that they may act as amplifying hosts for RRV and to the further suggestion that viraemic horses could transport RRV from peri-urban to urban or city environments (Doherty et al., 1966; Gard et al., 1977; Cloonan et al., 1982; McManus and Marshall, 1986; Pascoe et al., 1978). Apart from RRV, antibodies to BFV, MVEV, and Sindbis virus (SINV) have also been detected in horses (Table 1). In addition, isolation of West Nile virus Kunjin strain (KUNV) following experimental infection (Badman et al., 1984) and natural infection (Frost et al., 2012) indicate the possible role of horses in the transmission cycle of each of these viruses. KUNV was responsible for a large outbreak of neurological disease in horses in 2011 (Frost et al., 2012; Roche et al., 2013). While neutralising antibodies against neglected Australian arboviruses have been detected in cattle (Table 1), Kay et al. (1985b) were unable to detect viraemia in cows infected by MVEV using *Cx*. *annulirostris*. Intensive pig farming and a large feral pig population may have aided to recent Australian JEV outbreaks (Williams et al., 2022). The latter serve as amplifying hosts of JEV and MVEV, and their geographic range and large number (∼ 3.2 million) provide a latent risk of spillover to domestic piggeries and humans (Mackenzie and Smith, 2024). It is unknown how population dynamics and distribution of amplifying hosts influence arbovirus transmission.

#### 3.1.3 Cats and dogs

No viraemia sufficient to infect mosquitoes developed in small domestic animals such as dogs and cats following experimental infection with RRV or BFV through the bite of infected *Ae*. *vigilax* (Boyd and Kay, 2002). A relatively low antibody prevalence (∼ 10%) in serological survey of these animals suggested them less likely to be significant reservoirs of RRV, BFV and JEV.

#### 3.1.4 Bats

Bats have been associated with several zoonotic pathogens including viruses causing Ebola (filo virus), Lassa fever (Lassa virus) and COVID-19 (SARS-CoV-2). They are also suggested for having association with arboviruses. Fruit bats, also called flying foxes are found to have low viraemia to an experimental infection with RRV and JEV, but still were capable of infecting susceptible mosquitoes (Ryan et al., 1997; van den Hurk et al., 2009).

### 3.2 Birds

As early as 70 years ago, Anderson postulated that birds might be the primary reservoir of MVEV. The detection of anti-MVEV antibodies in many *Ciconiiformes* (storks) and *Pelecaniformes* (ibises, herons and pelicans) (Anderson, 1953; Anderson, 1954; Anderson et al., 1958; Doherty, 1964; Gard et al., 1976; Liehne et al., 1976; Boyle et al., 1983), and the high prevalence of anti-MVEV antibodies in birds (from 44% in adults to 96% in juveniles) after the MVEV epidemics of 1974–1975 (Marshall et al., 1982a) supported Anderson’s hypothesis. Galahs, sulphur-crested cockatoos, corellas, and black ducks produced MVEV viraemias with titres of 10^2^ to 10^6^ SMIC LD_50_/mL for 1–9 days following the bite of infected *Cx. annulirostris* mosquitoes (Kay et al., 1985b). With this viraemia in birds, approximately 10% of recipient *Cx. annulirostris* acquired virus infection.

Many avian species, particularly members of the family Ardeidae (herons, egrets, and allies), whose distribution overlaps with *Culex* mosquitoes, exhibit high prevalence of MVEV and JEV (Soman et al., 1977). Ardeids are considered the main vertebrate hosts of MVEV (Selvey et al., 2014b). As these avian species migrate to and across Australia (Guay et al., 2012), they share habitats with numerous other resident host species, such as cattle egrets (*Bubulcus ibis*) and feral pigs. The current role of migratory birds and feral pigs in the maintenance and transmission of arboviruses is a key health research priority for Australia. Where new wetlands, viraemic birds, and high mosquito densities converged near piggeries, the probability of “spillover” and rapid amplification in domestic pigs increased, causing the 2022 JEV outbreak in southern Australia. However, the mechanism of interaction between feral pigs, wildlife, and domestic animals is not clearly known.

The detection of antibodies to MVEV and KUNV in chickens (Doherty et al., 1968), was followed by isolation of MVEV from a sentinel chicken at Echuca, a town on the banks of the Murray River in northern Victoria, during the MVEV epidemic of 1974 (Campbell and Hore, 1975). Subsequently, the health department of several Australian state governments have employed flocks of sentinel chickens to monitor transmission of MVEV and KUNV as an early warning surveillance system to identify the threat of outbreaks (Doherty et al., 1976; Mackenzie et al., 1992). Besides MVEV, other arboviruses including Alfuy virus (ALFV), KUNV, RRV and SINV were also isolated from wild birds collected at Mitchell River Mission between 1963 and 1967 (Whitehead et al., 1968; Doherty, 1972; Doherty, 1977; Doherty et al., 1971). Ongoing research also suggests that birds may contribute to transmission dynamics of RRV and BFV, although their role in maintaining these viruses is still unclear (Vieira et al., 2023).

Furthermore, the increase in rainfall in southern Australia and the migration of water birds due to flowing inland rivers could lead to heightened activity of MVEV in certain areas (Selvey et al., 2014a). These findings underscore the significance of understanding the association of Australian arboviruses with water birds in the context of public health challenges and disease transmission dynamics.

## 4 Arboviruses and their arthropod vectors

### 4.1 Mosquitoes

Australia harbours a diverse mosquito fauna of more than 300 species (Webb et al., 2016). However, arboviruses have been recovered from only around 30 of these, mainly from species of *Aedes*, *Anopheles* and *Culex* mosquitoes (Webb et al., 2016). This includes several species and subgenera of *Aedes* that were reclassified by some authorities as belonging to the *Ochlerotatus* genus (Reinert, 2000). Isolation of a virus from a mosquito does not imply that it is competent to transmit the virus or that the mosquito plays a significant role in the transmission of that virus (Kain et al., 2022). A mosquito is called a competent vector when an arbovirus is isolated from it in its wild-caught stage; the mosquito can transmit the arbovirus to a host; and the mosquito itself is infected when feeding upon a viraemic host.

Some mosquito species such as *Anopheles annulipes*, *Cx. annulirostris* and *Cx. australicus* are cosmopolitan throughout Australia (Russell, 1998). KOKV, KUNV, MVEV and SINV were each first isolated from *Cx. annulirostris* collected at Mitchell River Mission in 1960 (Doherty R. L. et al., 1963). BFV was isolated from *Cx. annulirostris* in northern Victoria in 1974 (Marshall et al., 1982b). *Cx. annulirostris* is a freshwater mosquito species that is most active from spring to late autumn (Russell, 1995). Females are opportunistic feeders that readily take a blood meal from a wide variety of vertebrates, including humans, mammals, and birds, depending on host availability (Kay et al., 2007; Gyawali et al., 2019b), proliferate under optimal conditions, and are capable of dispersing more than 4 km per day (O’Donnell et al., 1992). *Cx. annulirostris* is also the primary vector of JEV in Australia. The virus was isolated from this vector during a JE outbreak in 1995 on Badu Island (Ritchie et al., 1997a), and later also on other islands of the Torres Strait (Hanna et al., 1999). The competence of the vector to virus was further demonstrated when JEV infecting a laboratory colony of *Cx. annulirostris* was transmitted to mice (van den Hurk et al., 2003) and to flying foxes (van den Hurk et al., 2009) via vector bite.

Limited understanding of the spatiotemporal importance of individual *Culex* species in transmitting endemic MVEV and emerging JEV is attributed to the lack of longitudinal vector and arbovirus surveillance. Other endemic mosquito species that may play a role in JEV maintenance in Australia include two recently established vectors with limited distributions – *Culex gelidus* has been implicated in previous Australian JEV outbreaks, whereas *Culex tritaeniorhynchus* is responsible for most of the JEV transmission in Asia (van den Hurk et al., 2022).

*Culex quinquefasciatus*, *Cx. sitiens*, *Ae. camptorhynchus*, *Ae. notoscriptus*, and *Ae. vigilax* are other common Australian mosquitoes (Russell, 1995). *Culex sitiens* is usually found around pools, puddles, ponds, wells, ditches, and rock pools, and often frequents tidal marshes and mangrove swamps. Females are primarily ornithophagic (i.e., feed on birds) but do feed on humans as well (Webb et al., 2016). *Culex quinquefasciatus* is active only during the warmer months, is generally ornithophagic and feeds on humans during the middle of the night (Webb et al., 2016). *Ae. vigilax* is a coastal saltmarsh mosquito that breeds in the brackish waters of mangrove swamps and salt marshes. Females are highly active at sunset, very aggressive biters and feed on humans and domestic animals (Belkin, 1962). The first isolate of RRV was taken from *Ae. vigilax* (Doherty R. et al., 1963). *Ae. notoscriptus*, a peri-domestic mosquito, is a competent vector of RRV and for these reasons it has been suggested that this species be considered more seriously in the context of urban transmission (Watson and Kay, 1998).

*Aedes aegypti*, a major global vector of DENV, may have been introduced into Australia in the early or mid-19th century (Mackenzie et al., 1996) and is now widespread throughout urban tropical north Queensland. Although this mosquito was widely distributed across south-east Queensland until the 1950s, it is now limited to an area bounded by Wondai in the south, Goomeri in the south-east and Charleville in the south-west (Queensland Health, 2015; Gyawali et al., 2016c).

*Aedes albopictus*, the Asian tiger mosquito, is also very able to transmit DENV and is distributed throughout the Torres Strait Islands to the north of Queensland (Ritchie et al., 2006). JEV was isolated from this vector in Malaysia and in Taiwan (Vythilingam et al., 1995; Weng et al., 1999; Su et al., 2014). Laboratory experiments in Australia demonstrated infection to Australian *Ae. albopictus* by feeding an infectious blood meal with virus titre 10^3.5^ TCID50/mL (Nicholson et al., 2014). The ability of infected *Ae. albopictus* transmitting JEV to hosts (i.e. laboratory weanling mice) has been demonstrated in Taiwan (Weng et al., 1997). Overall, findings of vector competence within and outside Australia strongly suggest *Ae. albopictus* as a potential vector for JEV. However, it is not apparent that this mosquito has played a role in any outbreaks in Australia to date.

The Australian arboviruses associated with different mosquito species, whether in terms of competence or evidence of experimental virus transmission, are presented in Table 2.

**TABLE 2:** Arbovirus vectors (mosquitoes) and their distribution in Australia.

Mosquito species	Distribution	Associated arbovirus(es)	Reference(s)
*Anopheles amictus*	NSW, QLD, WA	BFV, EHV, RRV, SINV	Doherty et al., 1979; Russell, 1995; van den Hurk et al., 2002
*Anopheles annulipes*	All states/territories	BFV, MVEV, RRV, SINV, TRUV	Doherty, 1972; Russell, 1995; van den Hurk et al., 2002
*Anopheles bancroftii*	NT, QLD, WA	MVEV, SINV	Russell, 1995
*Anopheles hilli*	WA, NT, QLD	RRV, SINV	Russell, 1995; Knope et al., 2016
*Anopheles meraukensis*	QLD, WA	EHV, SINV	Doherty R. L. et al., 1963; van den Hurk et al., 2002
*Aedes aegypti*	QLD	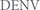	Knox et al., 2003
*Ochlerotatus alternans*	NSW, NT, QLD, SA, VIC, WA	RRV, SINV	Russell et al., 1991; Russell, 1995
*Ochlerotatus bancroftianus*	NSW, NT, QLD, SA, VIC, WA	BFV, EHV, GGV, RRV	Russell, 1995
*Ochlerotatus camptorhynchus*	NSW, SA, TAS, VIC, WA	BFV, KOKV, RRV, SINV	Marshall et al., 1982b; Ritchie et al., 1997b; Knope et al., 2016
*Ochlerotatus clelandi*	SA, TAS, VIC, WA	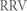	Russell, 1995
*Ochlerotatus flavifrons*	NSW, SA, TAS, VIC	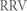	Russell, 1995
*Ochlerotatus funereus*	NSW, NT, QLD	BFV, 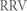	Lee et al., 1980; Russell, 1998; Ryan et al., 2000
*Ochlerotatus eidsvoldensis*	QLD, WA	BFV, GGV, MVEV, SINV	Russell, 1995
*Ochlerotatus procax*	NSW, QLD, VIC	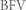 , 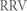	Kay and Standfast, 1987; Ryan and Kay, 1999
*Ochlerotatus lineatopennis*	QLD	RRV	van den Hurk et al., 2002
*Aedes multiplex*	NSW, southern QLD, VIC	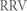	Ryan et al., 2000
*Ochlerotatus normanensis*	NSW, NT, QLD, WA	BFV, EHV, GGV, MVEV, RRV, SINV	Doherty et al., 1979; Broom et al., 1989; van den Hurk et al., 2002
*Ochlerotatus pseudonormanensis*	WA	BFV, MVEV, SINV	Sammels et al., 1999
*Ochlerotatus theobaldi*	NSW, QLD, SA, VIC, WA	GGV, RRV, SINV	Doherty, 1972
*Ochlerotatus tremulus*	WA	KUNV, MVEV, RRV, SINV	Liehne et al., 1981; Kay and Standfast, 1987
*Ochlerotatus sagax*	NSW, QLD, SA, VIC, WA	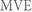 , RRV	Kay et al., 1989; Russell, 1998
*Ochlerotatus vigilax*	All states/territories	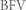 , EHV, GGV, KOKV, 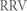 , SINV, STRV	Doherty R. et al., 1963; Kay et al., 1975; Boyd and Kay, 1999; Ryan et al., 2000
*Ochlerotatus notoscriptus*	NSW, NT, QLD, WA	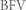 , 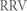	Ritchie et al., 1997b; Watson and Kay, 1998; Watson and Kay, 1999; Knope et al., 2016
*Coquillettidia linealis*	NSW, QLD, SA, VIC	BFV, EHV, GGV, RRV, TRUV	Russell, 1995; Jeffery et al., 2002
*Culex annulirostris*	All states/territories	ALFV, BCV, 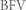 , EHV, EUBV, GGV, JEV, KOKV, KOOV, KOWV, 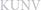 , MVEV, RRV, SINV, TRUV, WONV	Doherty et al., 1979; Kay et al., 1979; Kay et al., 1984; Kay et al., 1989; Ritchie et al., 1997b; Boyd and Kay, 2000; Ryan et al., 2000; van den Hurk et al., 2002; Colmant et al., 2016; Inglis et al., 2016
*Culex australicus*	All states/territories	KUNV, MVEV, 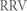 , SINV	Marshall et al., 1982b; Ryan et al., 2000
*Culex fatigans*	QLD	KUNV, SINV	Doherty et al., 1979
*Culex quinquefasciatus*	All states/territories	BFV, 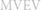 , RRV, SINV	Doherty et al., 1979; Kay and Standfast, 1987
*Culex palpalis*	WA	MVE, RRV	Russell, 1995
*Culex sitiens*	WA, QLD, NSW	BFV, KUNV, MVE, 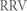 , SINV	Fanning et al., 1992; Boyd and Kay, 2000; van den Hurk et al., 2002
*Mansonia uniformis*	NSW, NT, QLD, VIC, WA	BFV, MVEV, 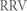	Russell, 1998; Ryan et al., 2000

NSW, New South Wales; NT, Northern Territory; QLD, Queensland; SA, South Australia; TAS, Tasmania; VIC, Victoria; WA, Western Australia. *Virus name abbreviations*: ALFV, Alfuy; BCV, Bunyip Creek; BFV, Barmah Forest; DENV, Dengue; EHV, Edge Hill; EUBV, Eubenangee; GGV, Gan Gan; JEV, Japanese encephalitis; KOKV, Kokobera; KOOV, Koongal; KOWV, Kowanyama; KUNV, West Nile virus Kunjin strain; MVEV, Murray Valley encephalitis; RRV, Ross River; SINV, Sindbis; STRV, Stratford; TRUV, Trubanaman; WONV, Wongal. *Note:* Dark grey shading: vector is competent to transmit the virus. Light grey shading: vector is poorly competent to transmit the virus. 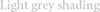: vector competence data are not available for the virus.

### 4.2 Ticks

Very little is known about tick-borne arboviruses in Australia (Dehhaghi et al., 2019). Approximately 70 species of ticks are found in Australia, 16 of which are known to feed on humans (Australian Government Department of Health and Aged Care, 2023). Upolu virus, a bunyavirus, was isolated from the widely distributed soft-bodied tick, *Ornithodoros capensis* on the Great Barrier Reef, in 1966 (Doherty et al., 1969). Nugget (Orbivirus) and Taggert (Nairovirus) are Kemerovo and Sakhalin group viruses and have been isolated from hard-bodied ticks (*Ixodes uriae*) from Macquarie Island (Doherty et al., 1975), in the Southern Ocean south-east of Tasmania. Saumarez Reef virus is a flavivirus that was isolated from *Or. capensis* and *Ix. eudyptidis* in the Australian region (St George et al., 1977). The transmission cycles and the importance of these viruses in human infection are unresolved.

### 4.3 Midges

Most of the viruses of the *Orbivirus* serological group (Bluetongue, Corriparta, Eubenangee, Palyam, Wallal and Warrego viruses) were isolated from biting midges such as *Culicoides brevitarsis* and *C.* marksi (Doherty et al., 1977; Standfast et al., 1984). There are reports of the alphavirus, BFV, replicating in, and being isolated from, *C. brevitarsis* and *C.* marksi (Standfast et al., 1984). However, it is not known if this vector is competent to transmit BFV. Another virus, Thimiri, from the Simbu sero-group, was isolated from *C. histrio* collected from northern Australia (Standfast and Dyce, 1982). The previous isolations of Thimiri virus were from birds in India and Egypt (Carey et al., 1971) but the vertebrate host in Australia is uncertain, and the role of these viruses in human infection is yet to be determined.

### 4.4 Sand flies and black flies

To date, no arbovirus that is indigenous to Australia has been identified to have a transmission cycle involving either a sand fly or a black fly. However, examples do exist of the *Phlebotomus* and *Simulium* genera providing competent vectors of arboviruses elsewhere in the world (Blair and Olson, 2015) – for sand fly-borne phleboviruses and black fly-borne rhabdoviruses, respectively (Kuno and Chang, 2005). Hence, these transmission routes should be considered alongside others for those many neglected Australian arboviruses for which our knowledge of their transmission epidemiology is incomplete.

## 5 Emerging public health threat

There is growing awareness among the Australian healthcare community that indigenous arboviral diseases have a serious impact on national public health (Taylor-Robinson, 2021). More widely, they pose a global epidemic risk (Gyawali et al., 2016d), as exemplified by RRV outbreaks across several Pacific islands (Aaskov et al., 1981; Rosen et al., 1981; Tesh et al., 1981). The projected escalation of human activity in the tropical north of Australia, including economic development and urbanisation, will bring humans into close contact with native reservoir wildlife and vector mosquitoes for Australian indigenous arboviruses (Gyawali and Taylor-Robinson, 2017). The expanded agriculture sector predicted for these locations will change the ecology of these mammals, birds, and insects (Gyawali et al., 2017b). Furthermore, unforeseen climatic and environmental variations (Inglis, 2009), such as increased incidence of cyclones, heavy rainfall, and resultant intensified flooding associated with outbreaks of RRV (Tall et al., 2014) and MVEV (Selvey et al., 2014b), have occurred of late with disconcerting regularity (Knutson et al., 2010), potentially effectuating an ecological change for Australian arboviruses. The projected future climatic suitability of Northern Australia for competent vector mosquito species needs to be evaluated. Moreover, it is worth noting that already this century close relatives of many of these neglected arboviruses have caused regional epidemics and global pandemics (Gyawali et al., 2016b; Mayer et al., 2017).

In recent years, the Australian Government has made significant efforts to develop the regional Australia, focusing northern tropical region of Australia in principle (Australian Government, 2015). As defined by the Northern Australia Infrastructure Facility Act 2016, this aims to harness water resources and improve trade, business, and transport infrastructure in order to stimulate employment and population growth in those historically underinvested area (Australian Government Department of Infrastructure, Transport, Regional Development, Communications and the Arts, 2024). One challenge of the federal government’s commitment to facilitating growth across regional Australia is the potential emergence of unique and poorly understood healthcare threats. With increased human activity in this remote and medically underserved region, there is a major risk of encountering neglected arboviruses that have not been extensively studied (Gyawali and Taylor-Robinson, 2017; Gyawali et al., 2017a).

The economic and social development of the currently sparsely populated tropical north of Australia is set to bring infection-naïve humans into close contact with native reservoir hosts and vector mosquitoes. This convergence of factors may precipitate an increased prevalence of infection with neglected indigenous arboviruses. Moreover, the escalating rate and effects of climate change that are increasingly observed in the tropical north of the country will likely drive a population boom of arbovirus-transmitting mosquitoes. As a commensurate response, continuing assiduous attention to vector monitoring and control is required, harnessing artificial intelligence to rapidly process large volumes of data, thereby improving data analysis, prediction (Sinclair et al., 2019), and decision-making (Taylor-Robinson, 2023). In this overall context, improved epidemiological surveillance and diagnostic screening, including establishing novel, rapid pan-viral tests to facilitate early diagnosis and appropriate treatment of febrile primary care patients, should be considered a public health priority.

## 6 Future directions

This brief article summarises our current understanding of the complex transmission dynamics between virus, vector and host for neglected Australian arboviruses. It is apparent that there are large knowledge gaps that need closed as a future research priority. Yet, already from the available information, including a deep dive into the sources cited here and detailed elsewhere, public health stakeholders across the nation should be exhorted to consider the complex transmission dynamics between virus, vector, and host for neglected Australian arboviruses.

Key questions to address include:

(1) What are the specific arboviruses indigenous to Australia that are associated with neglected diseases, and what is their prevalence among human populations?
(2) How do arboviruses interact with their arthropod vectors and vertebrate hosts in transmission cycles, and what factors influence the efficiency of transmission between these entities?
(3) Are there alternative modes of transmission for arboviruses, such as vertically, that may impact their circulation and persistence in nature?
(4) How do environmental factors, including climate change, urbanization, and land use changes, influence the transmission dynamics of arboviruses and their vectors in Australia?
(5) What is the role of different vertebrate reservoir hosts in the maintenance and amplification of arboviruses, and how does this impact the risk of spillover to humans?
(6) How do within-host dynamics, such as viraemia levels and host immune responses, affect the transmission success of arboviruses and their ability to establish infection in new hosts?
(7) What are the implications of co-circulation of multiple arboviruses in a given region on transmission dynamics, vector competence, and disease outcomes?
(8) How can advanced technologies, such as artificial intelligence and genomic analyses, be leveraged to enhance surveillance, prediction, and response measures for arbovirus disease outbreaks in Australia?

By shedding light on these interrelated, multifactorial issues researchers can gain a comprehensive understanding of which vector(s) and virus(es) represents a potential threat to Australian public health, and of which geographical location(s), region(s) or state(s) should be targeted for routine vector and virus surveillance and control. Only through unravelling the intricate interactions between viruses, vectors, and hosts in the transmission dynamics of neglected Australian arboviruses will effective strategies for disease control and public health interventions be achieved.

While arbovirus species that are indigenous to Australia provide the focus of this review it should be noted that the same principles broadly apply to invasive species, such as DENV and JEV, and potentially CHIKV and ZIKV, that are mentioned briefly in context herein. As all of these are important human pathogens, given their widening global distribution in recent times there is a growing need for outbreak preparedness. Similar research to that described above is required to determine the capacity for reservoir infections in Australia’s unique native animals and birds as well as the vector competence and ecology of the country’s mosquito species.

## 7 Conclusion

Extremely little is known about the distribution, epidemiology and transmission ecology of neglected arboviruses that are native to Australia. There is also scant information on the immunopathology and true disease burden, including undiagnosed acute undifferentiated febrile illnesses, for which they are a likely cause. This is despite their predicted emergence as human pathogens in the rapidly developing Northern Australia, thus posing a significant public health threat to that vast region (Gyawali and Taylor-Robinson, 2017), and potentially more so globally (Gyawali et al., 2016d). Consideration of these focal points coupled with improved diagnostic protocols, including the preparation of first-line screening tests for a panel of arboviruses, would help to counter this emerging and hitherto neglected threat to the health of traditionally underserved communities. Moreover, understanding better the vector competence of mosquitoes is crucial for predicting and managing the spread of arboviruses that pose a risk to humans or livestock (Kain et al., 2022). Combatting the vectors that are the most competent, identifying the widest ranging reservoir hosts, assessing the risk of transmission in different locations, and developing targeted control strategies will all directly inform Australian public health efforts as well as contribute to global arbovirus surveillance and control initiatives.
